# Obtainment of Flavonoid-Enriched Fractions from Maqui (*Aristotelia chilensis*) and Murta (*Ugni molinae*) Extracts via Preparative HPLC and Evaluation of Their Anti-Inflammatory Effects in Cell-Based Assays

**DOI:** 10.3390/antiox14050600

**Published:** 2025-05-16

**Authors:** Amador Alburquenque, Carolina Busch, Gabriela Gómez-Lillo, Alexander Gamboa, Camilo Perez, Nelson Caro Fuentes, Martin Gotteland, Lilian Abugoch, Cristian Tapia

**Affiliations:** 1 Department of Food Science and Chemical Technology, Faculty of Chemical and Pharmaceutical Sciences, University of Chile, Dr. Carlos Lorca Tobar 964, Independencia, Santiago 8380494, Chile; 2 Department of Nutrition, Faculty of Medicine, University of Chile, Av. Independencia 1027, Independencia, Santiago 8380453, Chile; 3 Department of Environmental Sciences, Faculty of Chemistry and Biology, University of Santiago de Chile, Avenida Libertador Bernardo O’Higgins nº 3363, Estación Central, Santiago 9170022, Chile; 4 Centro de Investigación Austral Biotech, Facultad de Ciencias, Universidad Santo Tomás, Avenida Ejército 146, Santiago 8370003, Chile

**Keywords:** polyphenols, maqui, murta, chitosan, pre-HPLC, ORAC, cytotoxicity, NF-κB, Nrf2, inflammatory bowel disease

## Abstract

Polyphenols exert anti-inflammatory and antioxidant effects by modulating cell signalling pathways and transcription factors involved in inflammatory bowel disease (IBD). However, their stability during digestion can be compromised. Polymer coatings like chitosan (-C) help preserve their stability. Maqui (*Aristotelia chilensis*) and murta (*Ugni molinae*) are rich in antioxidant and anti-inflammatory compounds. This work aims to obtain extracts (E) and blends (B) enriched in delphinidin and quercetin glucosides from maqui (Ma) and murta (Mu) crude extracts using preparative chromatography methodology (Prep-HPLC) and to evaluate their effectiveness through in vitro and cellular assays. HPLC-DAD analysis revealed a marked increase in phenolic compound concentration in the BEMaMu and BCMaMu extract blends. Total quercetin glycosides (TQG) increased by 11-fold, and total anthocyanins increased by approximately 8-fold compared to the fruit blend (BMaMu). BCMaMu exhibited a significantly higher ORAC value compared to the estimated additive mixture, suggesting a synergistic effect. No cytotoxicity was observed for BEMaMu, BCMaMu, and their chitosan-coated versions (BEMaMu-C and BCMaMu-C) in Caco-2 and HT29-MTX-E12 cells at concentrations of 0.1–50 mg/mL. Notably, only chitosan-coated BCMaMu inhibited NF-κB expression and activated Nrf2 in TNF-α-challenged Caco-2 cells at 0.1 and 0.5 mg/mL.

## 1. Introduction

Polyphenols have anti-inflammatory, anti-carcinogenic, and antioxidant properties that help to prevent chronic diseases, such as inflammatory bowel disease (IBD). IBD, including Crohn’s disease and ulcerative colitis, are chronic disorders characterized by inflammation of the gastrointestinal tract resulting from over-activation of the immune system. More than 190 risk factors associated with IBD have been identified, with the Western diet being among the top ten [1]. Polyphenols exert anti-inflammatory and antioxidant effects through modulating cell signalling pathways and transcription factors that are critical in the progression of IBD by modulating the gut microbiome, supporting symbiont growth while inhibiting pathobionts. A relevant aspect of the disease is the pronounced infiltration of the lamina propria by innate and adaptive immune cells, resulting in elevated levels of pro-inflammatory cytokines, including tumour necrosis factor α (TNFα), interleukin-1β (IL-1β), and interferon-γ (IFN-γ). From a nutritional perspective, polyphenols are poorly absorbed in the human gut and thus act as prebiotic substrates, promoting the growth and diversity of the gut microbiome. However, their gastrointestinal transit subjects them to enzymatic and physicochemical degradation, reducing the concentration and bioavailability of active compounds.

Therefore, it is possible that polyphenols administered as supplements may have greater bioavailability, considering that they are extracted from a complex dietary matrix [2,3].

There is extensive evidence supporting the beneficial effects of fruit and vegetable consumption, mainly attributed to their fibre and polyphenol contents. In particular, the concentration and purification of these compounds represent a rational strategy to enhance their functional properties. However, few comparative studies have addressed this issue, and to the best of our knowledge, no investigations have specifically focused on contrasting the effects of purified berry-derived extracts. In this context, further research into these purified fractions and their bioactive potential is warranted.

The present study is part of a broader project aimed at developing a nutritional supplement to prevent inflammatory bowel disease (IBD) through the site-specific action of antioxidant and anti-inflammatory compounds in the colon. Unlike conventional strategies that seek to maximize the systemic absorption of functional compounds, this research focuses on preserving and locally delivering bioactive compounds derived from two fruits with high concentrations of anthocyanins and flavonols: maqui (*Aristotelia chilensis* (*Mol.*) *Stuntz*) and murta (*Ugni molinae Turcz*), respectively. These compounds share the flavylium ion as their core molecular structure, whose stability is essential for maintaining antioxidant activity. However, this ion is particularly susceptible to degradation during gastrointestinal transit. Copigmentation with polysaccharides such as chitosan has been reported to protect the flavylium ion from nucleophilic attack by water [4], thereby enhancing its stability through intermolecular interactions [5]. Additionally, flavonol-rich colourants with a high copigment-to-pigment ratio exhibit remarkable stability [5,6]. At this stage of supplement development, the key nutraceutical ingredients are being characterized, with particular attention to the challenges associated with protection, antioxidant activity, and anti-inflammatory effects.

Recent studies have demonstrated that the nanoencapsulation of maqui extract in chitosan–triphosphate (CTPP-ME) and chenopodin–alginate (CHA-ME) nanocarriers significantly preserved ORAC antioxidant activity compared to non-encapsulated extracts, while also reducing the premature release of anthocyanins [7]. These findings support the current approach focused on designing a colon-targeted supplement using encapsulation strategies based on natural biopolymers.

The most suitable solvents for the extraction of phenolic compounds are methanol, ethanol, and acetone, with acetone being the least polar and methanol the most polar. Acetone allows for the extraction of mostly non-glycosylated phenols, while, in contrast, methanol and ethanol can extract both non-glycosylated and glycosylated compounds [8]. The total polyphenols extracted are higher when the organic solvent is mixed with water at a rate of 50%, due to the greater polarity of certain phenolic compounds (e.g., glycosylated flavonoids) [9,10]. Preparative high-performance liquid chromatography (prep-HPLC) allows for the enrichment of certain phenolic compounds of interest from crude extracts. The reversed-phase mode with C-18 columns is preferred, as it separates various solutes while using aqueous mobile phases and less-toxic organic solvents. The most commonly used solvents are binary mixtures with an organic mobile phase composed of methanol, acetonitrile, ethanol, or tetrahydrofuran (THF) and an aqueous mobile phase with or without the presence of acid modifiers such as acetic, formic, or trifluoroacetic acid [11]. Prep-HPLC also uses a gradient elution mode (linear or multi-step linear) and a UV-Vis absorbance detector in the wavelength region (λ) of 190 to 600 nm, which is typically absorbed by polyphenols [11].

Two important natural endemic sources of polyphenolic compounds in Chile are maqui and murta. Maqui has a high total polyphenol content (3116 ± 155 mg EAG/100 g DW) and antioxidant capacity (ORAC: 37,174 ± 1809 μmol TE/100 g DW) [12]. Among these compounds, flavonoids are known for their high antioxidant, anti-inflammatory, neuroprotective, and antitumoral properties [13,14,15]. The main flavonoid components present in this fruit are anthocyanins (22.6 ± 0.2 g/kg DW), while non-anthocyanin compounds (mainly phenolic acids) are present in concentrations of 3.11 ± 0.02 g/kg DW. The main anthocyanins in maqui include delphinidin-3-glucoside (22%), delphinidin-3,5-diglucoside (17%), delphinidin-3-sambubioside (17%), and cyanidin-3-sambubioside-5-glucoside (16%). Other flavonoids include ellagic acid (30%), myricetin-3-glucoside (20%), myricetin-3-galactoside (10%), and dimethoxy-quercetin (9%) [16]. Anthocyanins are extracted using acidic solutions, as the flavylium cation (i.e., the quinoidal base that accounts for the antioxidant capacity of the molecule) is stable at pH between 1 and 3. At pH higher than 4, anthocyanins take the form of carbinols and chalcones, the latter of which can undergo chemical degradation to produce phenolic acids [15,17]. For the extraction of anthocyanins from maqui berries, mixtures of acidified (0.1% HCl) ethanol/water (80:20) [7], acidified (0.1% HCl) methanol/water (80:20) [16], 70% methanol cleaned via AmberliteXAD-7 [18], and acidified methanol (0.1% HCl) [19] have been used, among others. Notably, the concentration of anthocyanins widely varies, depending on the geo-climatic conditions, period of collection, drying method, and solvents used to obtain the extract [11,19]. Accordingly, the concentration of total anthocyanins (TAC)—expressed in mg equivalents of cyanidin-3-glucoside/g for the maqui berry—has been reported to range between 8.0 and 35.0 mg/g [7,16,19,20,21,22,23,24,25].

Murta (*Ugni molinae* *Turcz*) is a wild berry from southern Chile that is rich in phenolic compounds and has high anti-inflammatory and antioxidant capacities, based on its oxygen radical absorption (ORAC) of 43,574 ± 1833 μmol ET/100 g DW [26,27,28], as well as a high content of total polyphenols (PTF): 3492 ± 121 mg EAG/100 g DW [12]. Murta contains three major categories of phenolic-derived compounds: phenolic acids (55.88 mg/100 g DW), flavonoids, and hydrolyzable tannins, where quercetin-derived compounds are the major constituents, corresponding to 75.4% of the identified compounds, with gallic acid being another predominant phenolic compound [29]. Murta berries have a low concentration of anthocyanins—mainly delphinidin-3-O-glucoside (73%)—when compared to maqui [30]. The highest total polyphenol concentration from murta was obtained using MeOH/H_2_O 60/40 (6.65 mg EAG/g DW) as a solvent, compared to 100% MeOH and 100% water [31].

This work aimed to produce mixtures of compounds with high antioxidant and anti-inflammatory capacities from crude maqui and murta extracts, as well as to evaluate their effectiveness through cell-based assays. To this end, we developed prep-HPLC methods to obtain an extract enriched in the delphinidin glucoside from maqui and another enriched in the quercetin glucoside from murta. Subsequently, we selected the mixture with the highest antioxidant activity (ORAC). Finally, we evaluated the selected mixture (alone or coated with chitosan) through a cytotoxicity test in the CACO-2 and HT29-MTX-E12 cell lines, as well as the anti-inflammatory and antioxidant capacity effects according to its effects on the NF-κB and Nrf2 signalling pathways, respectively, in Caco-2 cells. The results obtained support the comparative assessment of the therapeutic efficacy of natural berries, ethanolic extracts, and purified compounds as sources of functional ingredients. This comparison gains particular relevance when considering their formulation with biopolymers such as chitosan, which may improve compound stability and enable targeted bioactive release.

## 2. Materials and Methods

### 2.1. Plant Materials

Freeze-dried maqui (Ma) and murta (Mu) fruits were obtained from Isla Natura Ltda. (Chiloé, Chile), a company certified under FSSC 22000 (ISO 22000), and USDA Organic standards. Botanical verification was conducted based on morphological characteristics. The company maintains full traceability and sustainability from harvest to processing.

### 2.2. Chemical Reagents

The following reagents were obtained from Sigma-Aldrich (St. Louis, MO, USA): low-molecular-weight chitosan (LMW), with a degree of 75–85% deacetylation and a viscosimetric molecular weight of 269 kDa; cyanidin-3-glucoside hydrochloride; delphinidin-3-O-β-D-glucoside hydrochloride; quercetin-3-glucoside hydrochloride; solvent HPLC-grade acetonitrile (AcN); acetone (AcO); methanol (Met); ethanol (Et); isopropanol (Iso-PRO); sodium; 2,2′-azobis [2-amidinopropane] dihydrochloride (AAPH); and fluorescein, 6-hydroxy-2,5,7,8-tetramethylchroman-2-carboxylic acid (Trolox). All other reagents used were of analytical grade.

### 2.3. Methods

#### 2.3.1. Preparation of Maqui and Murta Extracts and HPLC Quantification of Anthocyanins and Quercetin Glycosides

Maqui extract (EMa) was prepared according to Andrade et al. [7]. Briefly, lyophilized maqui powder was dissolved in a mixture of acidified EtOH/H_2_O (0.1% HCl, *v*/*v*) in an 80:20 ratio (*v*/*v*). Subsequently, the mixture was homogenized and centrifuged. The supernatant was filtered under pressure and evaporated. The product obtained had a pH of 4.3–4.5 and was stored in the dark at 4 °C.

For the murta extract (EMu), lyophilized murta powder (3 g) was re-suspended in 30 mL of ethanol/water (4:1 *v*/*v*) acidified with 0.1% HCl. The mixture was homogenized with high shear in a DLAB Scientific Co., Ltd. (Beijing, China) Model D160^®^ mixer at 3000 rpm for 5 min. The suspension was centrifuged at 3000× *g* (Model Z323 Hermle labortechnik GmbH, Wehingen, Germany) for 7 min at 4 °C. The supernatant was filtered through 0.45 µm HAWP filters (Milipore^®^, Burlington, MA, USA) in Sartorius AG pressure filter holders at 4 Bar (Goettingen, Germany). Subsequently, the sample was concentrated in a rotary evaporator at 166 mBar at 55 °C for 30 to 40 min. The obtained extract was washed with 10 mL of MiliQ water, then frozen at −20 °C and dried via lyophilization (Model FD5508 ShinBio base Co., Ltd., Yangju, Republic of Korea) for 48 h. Finally, the lyophilized extract was stored in PP-50 mL falcon tubes with anti-UV sheets under a drying hood.

The HPLC quantification of anthocyanin and quercetin glycosides was carried out as described by Andrade et al. [7], based on the method of Genskowsky et al. [16] with minor modifications. In brief, 200 µL of fresh maqui extract and 1800 µL of 1% (*v*/*v*) acetic acid in water were mixed. Then, approximately 1.5 mL of the sample was filtered and added to an amber vial for analysis on HPLC in triplicate. Similarly, 20 mg of the freeze-dried murta extract was dissolved in 1.5 mL of a 1:1 mixture of 1% (*v*/*v*) acetic acid in water and acetonitrile. Subsequently, the solution was diluted in 20 mL of the same solvent and transferred to an HPLC vial. Then, 50 µL of the sample was injected into the HPLC. Samples were run in triplicate.

The analysis of anthocyanins was carried out using a Waters Alliance 2695 chromatograph equipped with a Purospher RP-18 column (4.6 × 250 mm with a particle size of 5 µm). Detection was carried out using a PDA 996 Waters photodiode array, scanning between 200 and 700 nm (Milford, CT, USA). The working flow was 1 mL/min, where the injection volume was 50 µL with a mobile phase A of acetonitrile, a mobile phase B of 1% (*v*/*v*) acetic acid in water, and a linear gradient according to the time in minutes and mobile phase A/B ratio (in %) as follows: 0 min, 0% A/100% B; 2 min, 0% A/100% B; 15 min, 20% A/80% B; 20 min, 30% A/70% B; 30 min, 40% A/60% B; 37 min, 40% A/60% B; 38 min, 0% A/100% B; and 40 min, 0% A/100% B.

A standard cyanidin-3-glucoside calibration curve (524 nm) was established using a stock solution of cyanidin-3-glucoside. An amount of 0.733 mg of standard was dissolved in 0.5 mL of 1% (*v*/*v*) acetic acid in water. Then, 0.2 mL of the stock was diluted in 2 mL of 1% (*v*/*v*) acetic acid in water and, from this last dilution, aliquots of 0.1 mL were taken (0.2, 0.4, 0.5, and 1 mL). These were transferred to 5 mL amber flasks, and the volume of 1 mL was completed with 1% (*v*/*v*) acetic acid in water. The method was linear with a correlation coefficient R^2^ = 0.9998. The variance test indicated that the intercept was not significant at 0 (*p* = 0.49); therefore, it passed through the origin. The relative standard deviation was less than 2.0% over the entire range of analytical concentrations.

A standard quercetin-3-glucoside calibration curve (354 nm) was established using a stock solution of quercetin-3-glucoside. An amount of 5.0 mg of standard was dissolved in 5 mL of 1% (*v*/*v*) acetic acid in water/acetonitrile (1:1). From this last dilution, aliquots of 0.1 mL were taken (0.08, 0.05, 0.03, 0.02, and 0.01 mL), which were transferred to 1 mL amber flasks, and the volume of 1 mL was completed with 1% (*v*/*v*) acetic acid in water/acetonitrile (1:1). The method was linear with a coefficient of determination (R^2^) greater than 0.9996. The variance test showed that the intercept was not significant at 0 (*p* = 0.14); therefore, it passed through the origin. The relative standard deviation was less than 2.0% over the entire range of analytical concentrations.

#### 2.3.2. Preparative Chromatography Conditions (Prep-HPLC)

Concentrated maqui extract and lyophilized powder obtained through the extraction of murta (see Section 2.3.1) were reconstituted at a concentration of 30 mg/mL using 1% *v*/*v* acetic acid in a single flask, shaken until the sample was completely homogeneous, and filtered at 0.45 um using a syringe filter (MCE, FMC201030-ZX, Darmstadt, Germany). Then, 3 mL of the sample was injected for analysis on a Buchi C-850 preparative chromatograph, Flawil, Switzerland (performed in triplicate).

The purified maqui and murta extracts were recovered on a Buchi C-850 preparative chromatograph using a C18 AQ column (250 × 20 mm, 5 µm particle size) (Flawil, Switzerland). The detection of anthocyanin glycosides from maqui extract and quercetin glycosides from murta extract was performed in preparative mode at 524 nm and 345–370 nm, respectively, with a high UV detection sensitivity (AU = 0.01). The most suitable chromatographic conditions were chosen to optimize the recovery of total anthocyanins from the maqui extract, as follows:

The workflow was set at 15 mL/min, while the injection concentration was kept constant at 30 mg/mL, with an injection volume of 5 mL. The equilibration time between each run was 15 min.

The solvents used were an acidified aqueous phase [A], and an organic solvent [B]. An isocratic gradient of 100% solvent [A] (composed of 1% *v*/*v* acetic or formic acid) was used for 5 min, followed by 100% organic solvent [B] (acetonitrile [ACN], methanol [Met], ethanol [Et], acetone [ACT] or isopropanol [iso-PRO]) for 25 min. The solvent mixture was selected through an experimental design, designated as experimental design 1. Once the solvent mixture was selected, the gradient was varied between 0 and 40% [A] for 5–10 min and between 60 and 100% [B] for 15–30 min. The gradient was chosen through another experimental design, designated as experimental design 2.

The optimal conditions for the recovery of quercetin glycoside from murta extract were selected as follows:

The workflow was set at 15 mL/min, while the injection concentration was kept constant at 30 mg/mL, with an injection volume of 3 mL. The equilibrium time between each run was 10 min.

The aqueous phase [A] was 1% *v*/*v* acetic acid solution, and the organic solvent [B] was selected from ACN, Met, Et, or iso-PRO for the solvent mixture. The gradient varied between 0 and 70% [A] for 0–5 min and from 70 to 100% [B] for 5–20 min. The organic solvent [B] and the gradient were chosen through another experimental design, designated as experimental design 3.

Quantification of total concentration of anthocyanins (TAC): TAC was expressed as ug/mL of cyanidin-3-glucoside for experimental design 1 (selection of optimal solvents) and as ug/mL of delphinidin-3-O-β-D-glucoside for experimental design 2 (optimal CPrep conditions for anthocyanin recovery). The TAC of the extracts obtained through C-prep was determined via UV-Vis spectrophotometry on a Jenway 6715 spectrophotometer (Stone, Staffordshire, UK) at a wavelength of 524 nm. Calibration curves were prepared from respective stock solutions using standards of cyanidin-3-glucoside (R^2^ = 0.999) for experimental design 1 and delphinidin-3-O-β-D-glucoside (R^2^ = 0.998) for experimental design 2.

Quantification of mass recovery of quercetin glycosides (QG): The concentration of murta extract obtained through C-prep was determined via UV-Vis spectrophotometry on a Jenway 6715 spectrophotometer at 354 nm. The calibration curve was obtained with the respective stock solutions using quercetin-3-glucoside as a standard (R^2^ = 0.999). The mass recovery of quercetin glycoside (QG) was estimated by multiplying the concentration by the volume recovered in each run, as these volumes were very variable. Therefore, the mass estimation reflects the effect of the experimental conditions tested.

#### 2.3.3. Experimental Designs

Experimental design 1. Two acids were evaluated for use as the acidified aqueous phase [A]: formic and acetic, both at 1% *v*/*v*; meanwhile, for the organic solvent [B], the following solvents were studied: ACN, Met, Et, ACT, and iso-PRO. For this purpose, a factorial model composed of 3 blocks, with 10 runs each, and a response variable (total anthocyanins concentration expressed as ug/mL cyanidin-3-glucoside equivalents) was designed.

Experimental design 2. From the previous design, acetic acid 1% *v*/*v* was selected as the acidified aqueous phase [A] and iso-PRO as the organic solvent [B], and the optimal gradient to be used was determined. For experimental design 2, a Box–Behnken design with six central points and quadratic processing was carried out, with total anthocyanins (µg/mL; expressed as delphinidin-3-O-β-D-glucoside equivalents) as a response variable. The model had four factors: A (solvent proportion [%A]), B (solvent proportion [%B]), C (solvent A elution time [min]), and D (solvent B elution time [min]). The gradient was managed between 0 and 40% [A] for 5–10 min and between 60 and 100% [B] for 15–30 min. The experimental design consisted of 30 random experiments whose samples were analyzed in triplicate.

Experimental design 3. For experimental design 3, a computer-designed experimental design followed by a 2-factor (2-F) interaction optimization model was used to determine the best recovery gradient for quercetin glycosides. The optimization model consisted of 29 randomized experiments. The aqueous phase [A] was composed of 1% *v*/*v* acetic acid, while the organic phase [B] was composed of ACN, Met, Et, or iso-PRO, in concentrations defined by the optimization model. The gradient was managed between 0 and 30% [A] for 0–5 min and between 70 and 100% [B] for 5–20 min.

#### 2.3.4. Determination of Optimal Maqui/Murta Mixture Obtained by Preparative HPLC

Mixtures of ingredients were made using the lyophilized powder of maqui and murta concentrates recovered via preparative HPLC. The mixture used for each experiment was obtained through the reconstitution of each ingredient in phosphate buffer (pH 7.4). An optimization model was designed for the mixtures, with A denoting maqui concentrate, expressed as a percentage of delphinidin 3-glucoside, and B denoting murta concentrate, expressed as a percentage of quercetin-3-glucoside. Each of the ingredients ranged from 0% to 100%, in such a way that they amounted to 100%. The antioxidant capacity ORAC (µmol ET/100 g) was used as a response variable. The proportions of A and B in the optimal mixture were obtained using the simplex-lattice method. The experimental design consisted of six combinations, each in triplicate (A/B 100/0; A/B 50/50; A/B 0/100; A/B 75/25; A/B 50/50; A/B 25/75) and the results were fitted to a linear model.

In the oxygen radical scavenging capacity (ORAC) analysis, ORAC was determined as previously described by Huang et al. [32], with some modifications according to Andrade [7].

#### 2.3.5. Coating of Mixtures of Extracts with Chitosan

A blend of maqui/murta extract (80/20 *w*/*w*; BEMaMu) and an optimal blend of maqui/murta concentrated via prep-HPLC (94.5/5.5; BCMaMu) were coated with chitosan as described by Tapia et al. [33]. Each mixture was manually mixed with a 1% *w*/*v* chitosan solution of acetic acid at a 1.25:1 ratio until a homogeneous paste was formed. The resulting paste was then freeze-dried and ground to a fine powder using a mortar and pestle. Finally, the powder was sieved through a #40 mesh AISI 316 stainless steel sieve (New Belin, WI, USA). The blends coated with chitosan were named BEMaMu-C and BCMaMu-C, respectively.

#### 2.3.6. Cell Assays

Cell culture: Caco-2 (catalogue number 86010202-1VL) and HT29-MTX-E12 (catalogue number 12040401-1VL) cell lines were cultured in DMEM F12 supplemented with 10% FBS, non-essential amino acids, and an antibiotic–antimycotic mixture in an incubator (Sanyo MCO-18AC, Sanyo Electric Co., Ltd., Osaka, Japan) at 37 °C and 5% CO_2_.

Cytotoxicity assays: The cytotoxicity of the extracts was determined using the “In Vitro Toxicology Assay Kit, Lactic Dehydrogenase based” (Sigma-Aldrich, Saint Louis, MO, USA) and CytoTox-ONE™ Homogeneous Membrane Integrity Assay (Promega Corp., Madison, WI, USA), according to the manufacturer’s recommendations [34,35]. Caco-2 and HT29-MTX-E12 cells were grown to confluence in 96-well plates. The cells were incubated with the samples at different concentrations (0, 0.1, 0.5, 1, 5, 10, and 50 mg/mL) for 1 h. For each experiment, a blank without cells was subtracted from the absorbance and/or fluorescence values. A positive control of cell damage was induced using 0.5% Triton X-100, representing 100% cytotoxicity. Each test was performed in triplicate.

Anti-inflammatory and antioxidant effects of the extract on NF-κB and Nrf2-transfected Caco-2 cells: Caco-2 cells were seeded at a density of 750,000 cells/mL in 96-well white plates. After 16 h, cells were transfected with the pNLF1-NRF2 (CMV/neo) or the pNL3.2.NF-kB-RE (NlucP/NF-kB-RE/hygro vectors) (both from Promega Corp., Madison, WI, USA) using TransIT(R)-2020 transfection reagent (Mirus Bio, Madison, WI, USA), according to the manufacturer’s recommendations. pKEAP1 (a regulator of intracellular expression of Nrf2) and pNRF2 plasmids were used in a 3:1 ratio at a final concentration of 10 ng/mL, and NFKβ plasmid was used at 10 ng/mL. After 24 h, the NRF2-transfected cells were incubated for 4 h with the extracts at concentrations of 0.5 and 1 mg/mL, while NFKβ-transfected cells were incubated with the extracts in the presence of TNFα (5 ng/mL). After incubation, luminescence was determined in each well using the Nano-Glo^®^ Luciferase Assay System reagent (Promega Corp., Madison, WI, USA). Cells incubated without extracts were used as negative controls, and Bardoxolone-methyl (MedChemExpress, Monmouth Junction, NJ, USA)—which acts as an inducer of NRF2 [36] and inhibitor of NFKβ [37,38]—was used as a positive control in both assays. Luminescence was measured using a Synergy HT^®^ instrument (Agilent BioTek, Santa Clara, CA, USA). Each test was performed in triplicate.

#### 2.3.7. Statistical Analysis

All results are expressed as means ± standard deviations. For experimental designs and total concentration quantification, data were obtained from three independent experiments (n = 3) and analyzed using Statgraphics^®^ Centurion XIX and IBM SPSS Statistics^®^ version 25. For cellular studies, results were obtained from three independent experiments (n = 2 per condition) and analyzed using GraphPad Prism^®^ version 8.3.0 (GraphPad^®^ Software, Inc., La Jolla, CA, USA). Levene’s test was used to assess the homogeneity of variance. When homogeneity was not met, Welch’s ANOVA was applied. Statistical differences between groups were evaluated using Tukey’s HSD (homogeneous variances) and Games–Howell (heterogeneous variances) post hoc tests (*p* < 0.05). For cellular assays, ANOVA was conducted at a significance level of *p* < 0.05 with a 95% confidence interval, followed by Bonferroni’s post hoc test for NF-κB and Dunnett’s post hoc test for Nrf2.

## 3. Results and Discussion

### 3.1. Determination and Optimization of Anthocyanin Recovery Conditions for Maqui Extract Using Preparative HPLC

According to the methodology described in Section 2.3.3, experimental design 1, a multi-factor analysis of variance was performed to compare the total anthocyanins concentration (TAC), expressed as cyanidin-3-glucoside equivalents (µg/mL). The factors of acidified aqueous phase [A] and organic solvent [B], as well as their interaction, had a significant effect on TAC (*p* < 0.0000). The multiple range test for mobile phase A showed a significant difference between 1% *v*/*v* acetic acid (37.6 µg/mL cyanidin-3-glucoside) and 1% *v*/*v* formic acid (33.0 µg/mL cyanidin-3-glucoside).

**Figure 1:**
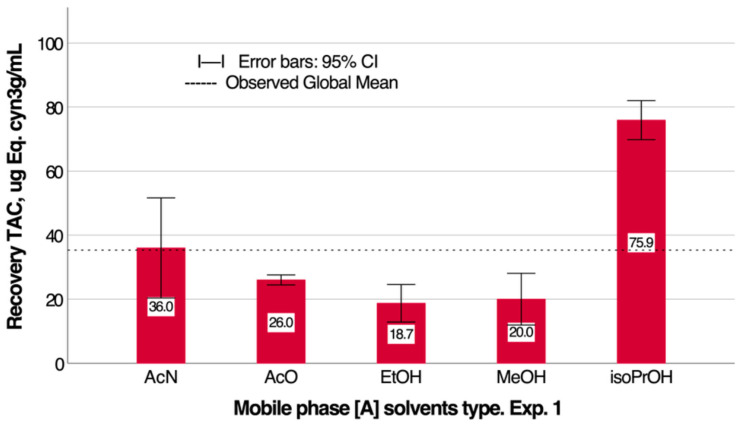
Recovery of total anthocyanin content (TAC), in µg Cyan-3-g/mL, using organic solvents: least squares means with 95.0% confidence intervals.

Figure 1 presents the recovery of total anthocyanin content (TAC) obtained using different organic solvents, expressed as least squares means with 95.0% confidence intervals. This figure provides a comparative overview of the extraction efficiency achieved with each solvent, highlighting the variations in TAC yield and supporting the selection of the optimal solvent for subsequent experiments. Regarding organic solvent B (see Figure 1), iso-PRO (75.9 µg/mL cyanidin-3-glucoside) showed the highest TAC recovery, followed by ACN (36.0 µg/mL cyanidin-3-glucoside) and AcO (26.0 µg/mL cyanidin-3-glucoside), while Et (18.7 µg/mL cyanidin-3-glucoside) and Met (20.0 µg/mL cyanidin-3-glucoside) showed the lowest recoveries. No significant differences between these solvents were observed.

Log *p* describes the solubility of a compound in the polar (aqueous) or non-polar (octanol) solvent. This parameter allows for the selection of the most suitable solvent to recover the analyte(s) of interest. Similar log *p* values between the solvent and the analytes result in higher solubility of the latter and, therefore, more significant recovery [39]. We sought the estimated log *p* values (XLOGP3-AA) for the solvents and the log *p* values (alogPS) for the main anthocyanins described for the maqui extract. The computed octanol–water partition coefficients (XLOGP3-AA) were retrieved from the PubChem database [40]. The log *p* values of the organic solvents [B] were as follows: methanol, −0.5; ethanol and acetone, −0.1; acetonitrile, 0; isopropanol, 0.3. Genkowsky et al. [16] have reported the polyphenolic profile of maqui extracts determined through an HPLC analysis of maqui berry—where anthocyanins represent the main concentration (42.4 g/kg dried mass) in contrast to non-anthocyanin compounds (3.1 g/kg dried mass). The anthocyanins identified, according to the retention time in minutes, were delphinidin-3-sambubioside-5-glucoside (9.92), delphinidin-3,5-diglucoside (9.95), cyanidin-3-sambubioside-5-glucoside (10.51), cyanidin-3,5-diglucoside (11.86), delphinidin-3-sambubioside (12.66), delphinidin-3-glucoside (13.25), cyanidin-3-sambubioside (14.49), and cyanidin-3-glucoside (14.86). The most significant component was delphinidin-3-glucoside.

The logP estimated from aLogPS, retrieved from the Food Database [41] for the anthocyanins identified by Genkowsky et al. [16], were as follows: delphinidin 3-sambubioside-5-glucoside, −0.63; delphinidin-3,5-diglucoside, −0.28; cyanidin-3-sambubioside-5-glucoside, no information; cyanidin-3,5-diglucoside, −0.18 [41]; delphinidin-3-sambubioside, 0.08; delphinidin-3-glucoside, 0.93; cyanidin-3-sambubioside, 0.33 [42]; and cyanidin-3-glucoside, 0.98.

According to the results obtained through experimental design 1, the highest recovery of total anthocyanins was obtained with isopropanol (0.3), followed by acetonitrile (0); these two solvents were those that presented the highest log *p* values. If we observe the log *p* values for delphinidin and anthocyanin glycosides, we see that only delphinidin-3-sambubioside (0.08), delphinidin-3-glucoside (0.93), cyanidin-3-sambubioside (0.33), and cyanidin-3-glucoside (0.98) presented the highest log *p* values. Therefore, the recovered anthocyanins probably correspond to these compounds. Genkowsky et al. [16] also indicated that the predominant compound in the maqui extract corresponded to delphinidin-3-glucoside, which would explain the higher recovery of anthocyanins with these solvents.

**Figure 2:**
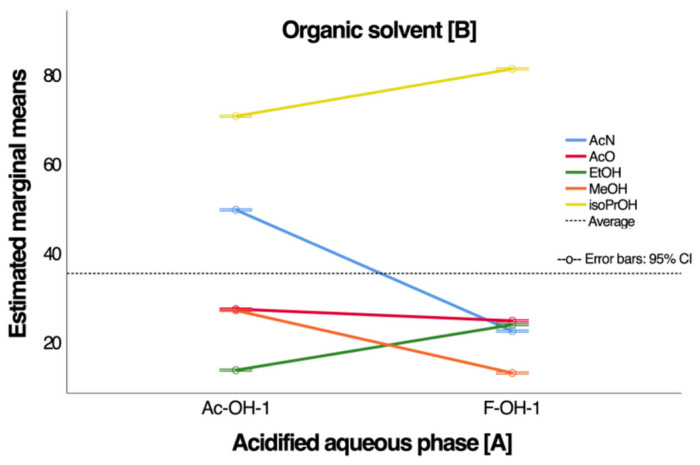
Interaction effects of aqueous and organic phases on total anthocyanin content (TAC) recovery.

In the interaction graph between the aqueous phase [A] and organic solvent [B], as shown in Figure 2, a strong interaction between the phases was observed. Iso-PRO had the highest TAC recovery with acetic acid (Ac-OH-1) and formic acid (F-OH-1). In the case of acetic acid, it was possible to differentiate the effect of the organic solvent [B] on the recovery of TAC. In contrast, for formic acid, only differences were distinguished between the maximum recovery with Iso-PRO and the minimum recovery with Met. An additional ANOVA analysis of the factors (A: data blocks, B: acidic aqueous solvents (Ac-OH-1% and F-OH-1%), and C: organic solvents) demonstrated that only the organic solvent elution phase had a statistically significant impact (*p* = 0.000). Therefore, acetic acid 1% *v*/*v* was selected as the acidified aqueous phase [A], and iso-PRO was selected as the organic solvent [B] for experimental design 2.

**Figure 3:**
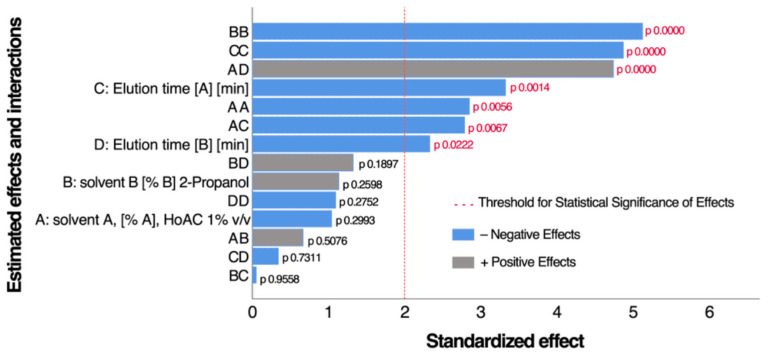
Standardized Pareto diagram for total anthocyanin content (TAC) at 524 nm (µg delph-3-glu/mL).

Experimental design 2, as described in Section 2.3.3, was utilized to estimate the optimal conditions for the recovery of total anthocyanins content (TAC), expressed in delphinidin-3-glucoside equivalents, which is likely one of the main anthocyanin components. Figure 3 presents the standardized Pareto diagram for TAC at 524 nm, showing the factors with the greatest standardized effects. Grey bars represent factors with a positive contribution to the response variable, while blue bars indicate negative effects. The vertical line marks the threshold for statistical significance. A Pareto diagram indicated seven significant effects (see Figure 3): BB, CC, AD, C, AA, AC, and D (in decreasing order). The fitted model explained 56.7% of the variability in TAC. We defined the operating conditions by maximizing the TAC value, which were as follows: solvent A [%A], HoAC 1% *v*/*v* = 0; solvent B [%B], 2-Propanol = 77; elution time [A] [min] = 7.8; and elution time [B] [min] = 15.

### 3.2. Determination and Optimization of TQG Recovery Conditions for Murta Extract Using Preparative HPLC

We defined the optimal chromatographic conditions for the recovery of TQG from murta extract through experimental design 3, as detailed in Section 2.3.3. The model included a categorical factor C1 (organic solvent [B]) and four quantitative factors: C2 (solvent proportion [%A]), C3 (solvent proportion [%B]), C4 (solvent A elution time [min]), and C5 (solvent B elution time [min]).

**Figure 4:**
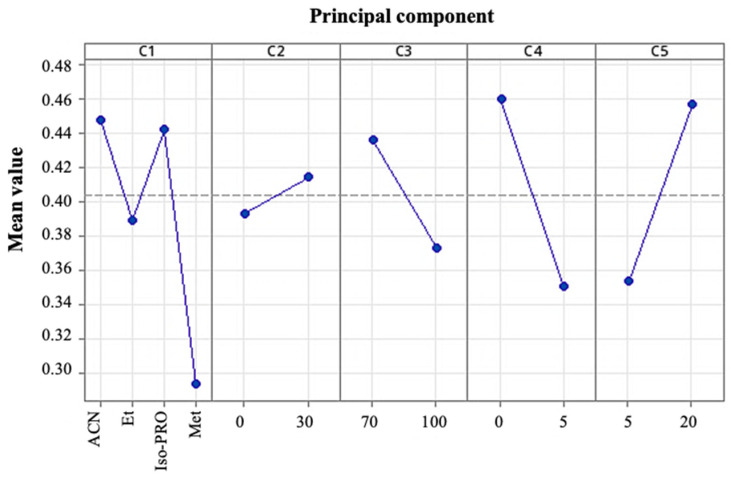
Principal component analysis for the five factors studied concerning the recovery of total quercetin glycosides (TQG)—C1 (organic solvent [B]) and four quantitative factors: C2 (solvent proportion [%A]), C3 (solvent proportion [%B]), C4 (solvent A elution time [min]), and C5 (solvent B elution time [min]).

**Figure 5:**
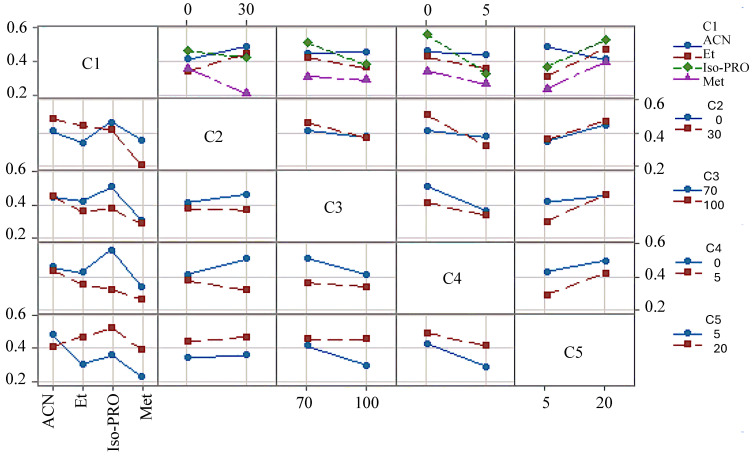
Interactions between the five factors studied concerning the recovery of total quercetin glycosides (TQG)—C1 (organic solvent [B]) and four quantitative factors—C2 (solvent proportion [%A]), C3 (solvent proportion [%B]), C4 (solvent A elution time [min]), and C5 (solvent B elution time [min]).

Figure 4 shows the results of a principal component analysis for the five factors studied concerning the amount of TQG recovered. According to these results, without considering the interactions between factors, the most suitable chromatographic conditions were found to be as follows: organic solvent [B], ACN or iso-Pro; proportion of solvent A, 30%; the proportion of solvent B, 70%; the elution time of A, 0 min; and the elution time of B, 20 min. Figure 5 depicts the interactions between factors and their effects on the recovery of TQG. The interactions with the other factors were considered when selecting between ACN and iso-Pro. According to Figure 5, the highest recovery of TQG was obtained for iso-Pro under the following conditions: proportion of solvent B = 70%; elution time of A = 0 min; elution time of B = 20 min. A slight decrease was observed compared to ACN under solvent proportion, A = 30%. Accordingly, iso-Pro was selected as the organic solvent of the mobile phase.

### 3.3. Determination of Optimal Maqui/Murta Concentrate Mixture Obtained via Preparative HPLC

**Figure 6:**
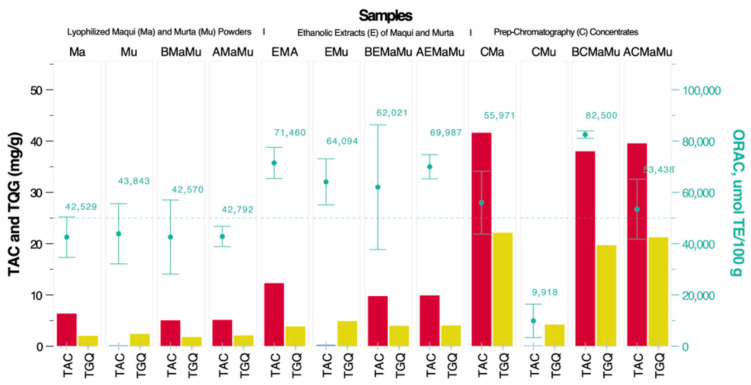
Total anthocyanin content (TAC), total quercetin glycosides (TQG), and antioxidant capacity (ORAC) in maqui, murta, and their extracts and preparative chromatography concentrates, enabling evaluation of potential synergistic effects.

The optimal maqui (CMa)/murta (CMu) concentrate mixture was determined according to the methodology described in Section 2.3.4. Table 1 shows the results for the six combinations used in the experimental design and the optimal mixture estimated using the simplex-lattice method. The response variable was the average ORAC value, expressed as µmol Trolox equivalents per 100 g of purified extract obtained via Prep-HPLC. The data fitted to a linear model (*p* = 0.0276) explained 74.2% of the variability in ORAC. Analysis of variance (ANOVA, *p* < 0.0001) and Tukey’s post hoc test indicated significant differences among the formulations. The maximum ORAC value (82,500 ± 578 µmol ET/100 g) was obtained for the optimized mixture consisting of 94.5% maqui concentrate (CMa) and 5.5% murta concentrate (CMu) and exhibited significantly higher antioxidant capacity compared to all other tested combinations (*p* < 0.001), including the pure individual formulations. These results indicate a synergistic interaction between the bioactive compounds, yielding an antioxidant effect that exceeds the expected additive response. Figure 6 illustrates the calculated additive ORAC effect based on the individual extract compositions, including the natural berry mixture (BMaMu), the berry ethanolic extract mixture (BEMaMu), and the purified extract mixture (BCMaMu). Furthermore, sample Exp4 also demonstrated a synergistic effect, with a statistically significant difference (*p* 0.036). Although a linear fitting model was applied, the observed interactions between components suggest an enhancement of antioxidant activity beyond linear additive predictions.

The ORAC value obtained for the maqui concentrate (CMa/CMu 100/0, 55,971 µmol TE/100 g) was similar to the maximum values reported in the Chilean antioxidant database [12] (47,260 µmol TE/100 g). However, the murta concentrate (CMa/CMu 0/100, 9918 µmol ET/100 g) was significantly lower than that reported in the same database (43,574 µmol ET/100 g). This difference can be attributed to the chromatographic conditions, which sought to optimize the recovery of delphinidin-3-glucoside in the case of the maqui concentrate and quercetin glucoside in the case of the murta concentrate. In any case, the ORAC value obtained for the optimized mixture (CMa/CMu 94.5/5.5%) was significantly higher (82,500 µmol TE/100 g) than that estimated for the additive maqui and murta concentrate mixture, indicating a synergistic effect.

It has been demonstrated that certain flavan-3-ols can recycle anthocyanins, such as malvidin-3-glucoside and peonidin-3-glucoside, by donating a hydrogen atom to their radicals generated through hydrogen abstraction. This process allows anthocyanins to return to their active form, thereby prolonging their antioxidant effects [43]. Likewise, protective and synergistic effects of polyphenols have been observed [44], which could be involved in the interactions between the compounds purified from maqui and murta. In addition, anthocyanins such as cyanidin-3-glucoside, in conjunction with other antioxidants, can inhibit oxidative enzymes through hydrophobic interactions and hydrogen bonds, thus enhancing their antioxidant efficacy [45]. Different anthocyanins may also act in a complementary manner, protecting each other from degradation and prolonging their antioxidant properties, as observed for the anthocyanin petunidin and the carotenoid lycopene, with respect to the Akt/Nrf2 pathway [46]. Finally, the presence of phenolic acids—which are particularly abundant in murta—could have favoured the regeneration of antioxidant radicals and improved the solubility of the active compounds, optimizing their activity in the ORAC test [47].

Figure 6 shows the concentrations of TAC, TQG, and ORAC values in maqui, murta, and their extracts and Prep-HPLC concentrates. The concentrations of TAC and TQG in maqui significantly increased after extraction (EMa) and subsequently after concentration via pre-HPLC (CMa), when compared to the freeze-dried berry (Ma). The highest ORAC value was observed with the extract (EMa) and not for the concentrate via pre-HPLC (CMa), suggesting the loss of other phenolic compounds with antioxidant activity. The concentrations of TAC and TQG in murta were lower than those reported in maqui, and there was a higher proportion of TQG than TAC. No significant differences were observed between EMu and CMu. ORAC activity was highest in the extract (EMu) and lowest in the subsequently concentrated extract (CMu), when compared with the freeze-dried murta (Mu), indicating a significant loss of other polyphenols with antioxidant activity. Subsequently, mixtures with different concentrations of TAC and TQG were evaluated, including maqui/murta 80/20 (*w*/*w*) for freeze-dried berry (BMaMu), ethanolic extracts (BEMaMu), and the mixture of concentrated extracts via pre-HPLC in a ratio of Maqui/Murta 94.5/5.5 (*w*/*w*) (BCMaMu). To evaluate possible synergy between the different components, TAC, TQG, and ORAC values were estimated, weighting for the percentage of each component in the mixture (AMaMu, AEMaMu, and ACMaMu). BEMaMu and BCMaMu did not show significant differences in TAC and TQG, compared to the additive mixture. However, for the BMAMu mixture, the TQG value was significantly lower compared to AMaMu. No differences in ORAC activity were observed between BMaMu and BEMaMu when compared to the additive mixture. However, a significantly higher ORAC value was observed for the mixture of maqui and murta concentrates (BCMaMu; ORAC: 82,500 µmol ET/100 g), compared to that estimated for the additive mixture (ACMaMU; ORAC: 53,438 µmol ET/100 g). Therefore, the only mixture that showed a synergistic effect concerning its ORAC value was that of maqui and murta concentrated via pre-HPLC (BCMaMu).

**Figure 7:**
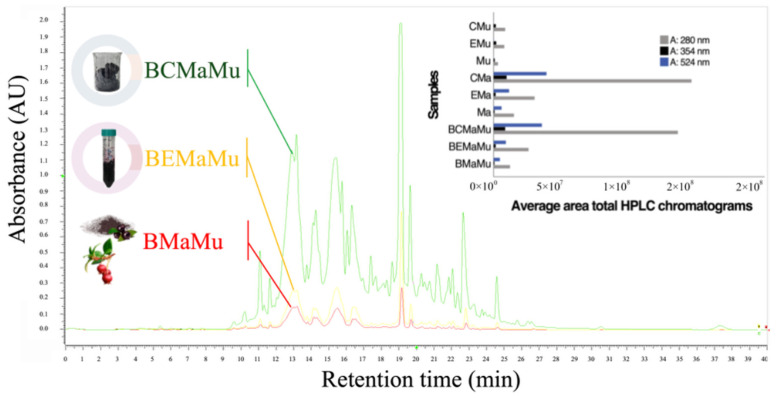
Overlay of HPLC-DAD chromatograms at 280 nm with superimposed area comparison and bar graph representation at 280, 354, and 524 nm.

**Figure 8:**
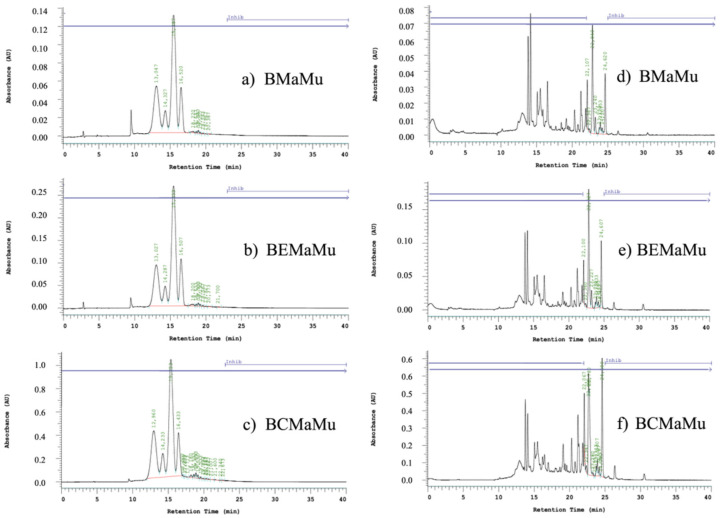
Comparative HPLC-DAD Chromatograms at 524 nm for Total Anthocyanin Content (TAC) and at 354 nm for Total Quercetin Glycosides (TQG) in BMaMu (**a**,**d**), BEMaMu (**b**,**e**), and BCMaMu (**c**,**f**) Extracts.

Figure 7 illustrates the overlay of HPLC-DAD chromatograms at 280 nm, along with a graphical representation of the total areas at 280 (total phenols), 354 (quercetins), and 524 nm (anthocyanins) for the natural berry mix (BMaMu), ethanolic extracts (EMaMu), and purified extracts (ECMaMu), indicating the progressive concentration of phenolic compounds across each treatment, which synergistically enhanced the antioxidant response, as shown in Figure 4, Figure 5 and Figure 6. For accurate quantification, the concentrations of total anthocyanins (TAC) and quercetins (TQG) were determined in subsequent analyses (see Figure 8).

Following the methodology outlined in Section 2.3.1, the concentrations of TAC and TQG in the samples were significantly increased. Figure 8 presents representative chromatograms for the same samples as those shown in the overlay (BMaMu, BEMaMu, and BCMaMu). The chromatograms display the identified peak areas at 524 and 354 nm, corresponding to TAC (glycosylated delphinidin) and TQG (quercetin-3-O-glucoside) equivalents, respectively. These compounds were identified through comparison with glycoside standards, providing a clear visualization of the targeted compounds in each sample.

The quantification chromatograms of maqui anthocyanins display a characteristic profile, as previously reported [16,48]. The analysis revealed a progressive increase in concentration, which aligns with the systematic purification processes applied, advancing from BMaMu to BEMaMu and ultimately to BCMaMu. The TAC and TQG concentrations in EMa and EMu are well documented in the literature, and the results obtained in this study are in agreement with previous reports [22,23,25,49]. In contrast, the purified extracts and blends (CMa, CMu, and CMaMu) exhibit significantly higher concentrations compared to conventional extracts, due to the purification processes conducted via preparative HPLC. For BMaMu, TAC concentrations in the BEMaMu and BCMaMu samples increased by 2- to 8-fold, while the TQG concentration increased by 2- to 11-fold, respectively.

### 3.4. Cell Assays

#### 3.4.1. Cytotoxicity Assays

**Figure 9:**
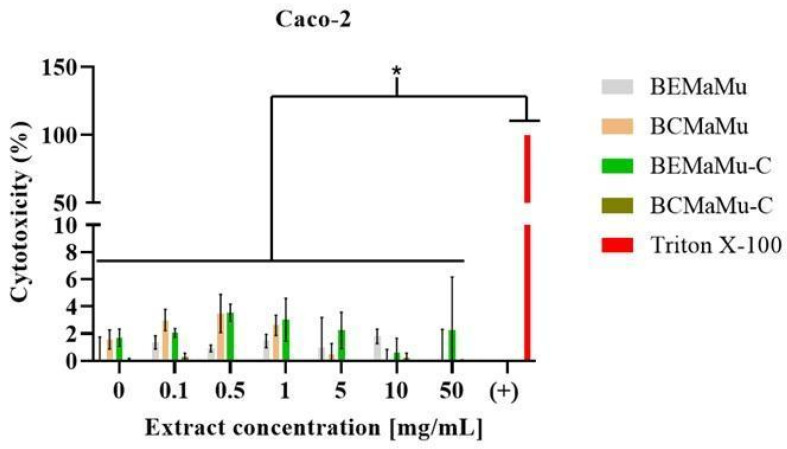
Cytotoxicity of the extracts in Caco-2 cells. Cells were exposed to the extracts (BEMaMu, BCMaMu, BEMaMu-C, and BCMaMu-C) in increasing concentrations. Triton X-100 was a positive control, which induced 100% cell death and LDH release. (*) indicates significant differences at *p* < 0.001 compared to the positive control.

**Figure 10:**
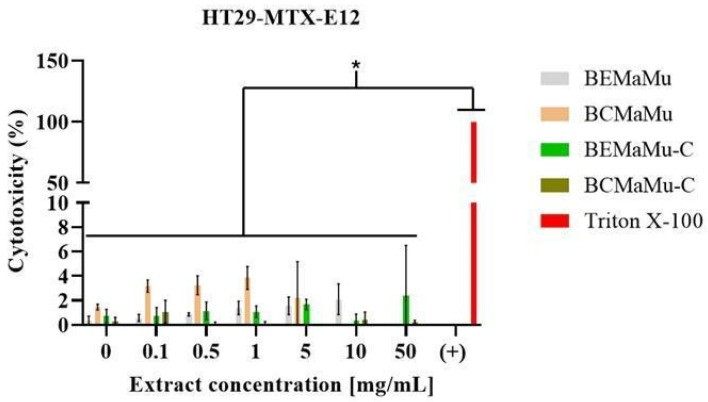
Cytotoxicity of the extracts in HT29-MTX-E12 cells. Cells were exposed to the extracts (BEMaMu, BCMaMu, BEMaMu-C, and BCMaMu-C) in increasing concentrations. Triton X-100 was a positive control, which induced 100% cell death and LDH release. (*) indicates significant differences at *p* < 0.001 compared to the positive control.

The cytotoxicity of the extracts (BEMaMu and BCMaMu) and the chitosan-coated extracts (BEMaMu-C and BCMaMu-C) was evaluated in vitro in Caco-2 and HT29-MTX-E12 cells (Figure 9 and Figure 10, respectively) through the measurement of LDH release. For each extract, cytotoxicity is expressed as the percentage of LDH release induced by cell exposure to 0.5% Triton X-100 (considered as 100% cytotoxicity). No significant cytotoxicity of the extracts was detected in both cell types, including at the highest concentration tested (50.0 mg/mL). According to ISO 10993-5:2009 [50], a compound is considered cytotoxic when it reduces cell viability by more than 30%, which was not observed in these studies. Consequently, the extracts and chitosan-coated extracts may be considered safe for use in humans, making their use in future human studies possible [51].

#### 3.4.2. Anti-Inflammatory and Antioxidant Effect of the Extracts in NF-κB- and Nrf2-Transfected Caco-2 Cells

**Figure 11:**
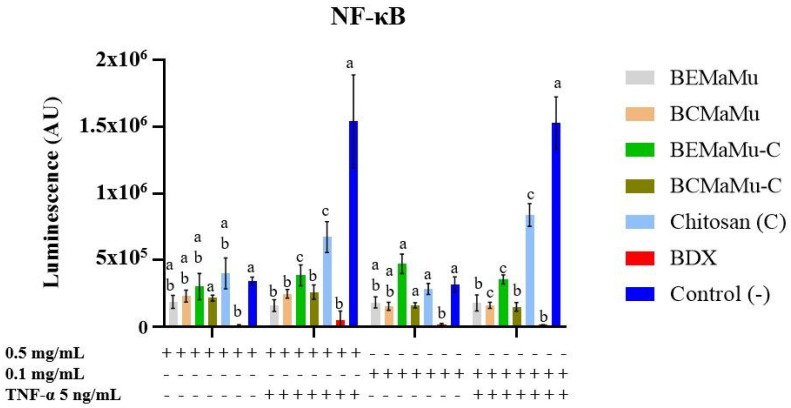
Anti-inflammatory activity of the extracts in NF-κB-transfected Caco-2 Cells. Different superscript letters indicate statistically significant differences at *p* < 0.0001.

As shown in Figure 11, TNF-α significantly increased (*p* = 0.0001) luminescence levels when compared to unstimulated cells, thus indicating NF-κB activation. Bardoxolone—an NF-κB inhibitor—not only prevented the TNF-induced increase in luminescence levels (*p* < 0.0001) but also decreased the luminescence levels observed in the cells not exposed to TNF-α. This latter observation suggests that a constitutive basal activation of NF-κB exists in these cells. In the absence of TNF-α, the extracts did not affect the luminescence levels, indicating that they had no impact on basal NF-κB activation in Caco-2 cells. In TNF-stimulated cells, the extracts prevented—either partially (BEMaMu-C and chitosan) or totally (BEMaMu, BCMaMu and BCMaMu-C)—the increase in luminescence induced by TNF, when compared to BDX. Therefore, BEMaMu, BCMaMu, and BCMaMu-C appear to be the most efficient in generating anti-inflammatory effects. Interestingly, chitosan exerted a certain anti-inflammatory activity.

**Figure 12:**
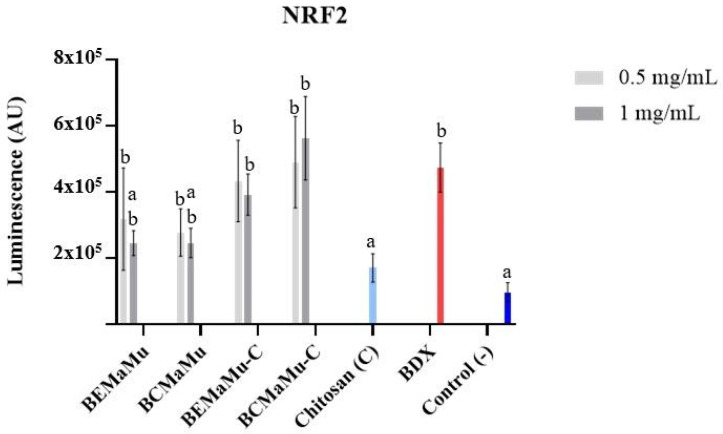
Effect of extracts and chitosan coating on Nrf2 activation. Different superscript letters indicate statistically significant differences at *p* < 0.0001.

The antioxidant properties of the extracts were determined in Nrf2-transfected cells. Nrf2 is a transcription factor involved in the maintenance of cellular redox homeostasis during increased oxidative stress. As shown in Figure 12, BDX significantly increased (*p* = 0.0001) the luminescence levels, compared to the negative control, reflecting the activation of Nrf2 by this agent in Caco-2 cells. Compared to the negative control, BEMaMu, BCMaMu, and chitosan did not affect the luminescence levels. At the same time, BEMaMu-C and BCMaMu-C increased the luminescence compared to the negative control (similarly to BDX), indicating that these extracts can activate Nrf2. The observed effect for each of the extracts did not vary according to their concentrations. The results with chitosan alone contrast with other studies reporting Nrf2 activation with this compound; for example, in neuronal cells exposed to hydrogen peroxide [52].

Furthermore, an in silico study suggested that chitosan would activate NRF2 due to the strong binding of chitosan to KEAP-1 and the subsequent inhibition of Keap1-NRF2 interaction [53]. These differences from our results could be due to the use of high-molecular-weight chitosan in their study, while we used LMW chitosan. These results suggest that the increase in luminescence levels observed for the chitosan-coated extracts was not directly due to chitosan [54,55].

The protective anti-inflammatory and antioxidant effects observed in this study are probably due to specific flavonoids in the extracts; for example, we have previously reported that quercetin (present in murta) prevented NF-κB activation and prevented the decrease in nuclear translocation of Nrf2 induced by indomethacin in rats and Caco-2 cells [56]. On the other hand, another study using cyanidin-3-glucoside (present in maqui) in Caco-2 cells exposed to TNFa also reported inhibition of the NF-KB signalling pathway and activation of Nrf2 [57]. Therefore, our current results align with those of previous studies.

## 4. Conclusions

The optimal recovery conditions of TAC from maqui extract (EMa) using preparative HPLC were acetic acid 1% *v*/*v* for the acidified aqueous phase [A] and iso-PRO for the organic solvent [B]. The operating conditions were as follows: solvent A [%A], HoAC 1% *v*/*v* = 0; solvent B [%B], 2-Propanol = 77; elution time [A] [min] = 7.8; elution time [B] [min] = 15. For the recovery of TQG from murta extract (EMu), the optimal operating conditions were as follows: solvent A [%A], HoAC 1% *v*/*v* = 0; solvent B [%B], 2-Propanol = 70; elution time [A] [min] = 0; elution time [B] [min] = 20. The optimal maqui/murta concentrate mixture obtained via preparative HPLC (BCMaMu) was determined in a ratio of 94.5/5.5%, based on the obtained ORAC value (82,500 µmol ET/100 g). This mixture presented a synergistic effect, as the ORAC value estimated for the additive mixture was 53,438 µmol ET/100 g. HPLC-DAD analysis revealed a marked increase in phenolic compound concentration in the BEMaMu and BCMaMu extract blends. Total quercetin glycosides (TQG) increased by 11-fold, and total anthocyanins increased by approximately 8-fold compared to the fruit blend (BMaMu).

No cytotoxicity was detected for the extracts (BEMaMu and BCMaMu) and the chitosan-coated extracts (BEMaMu-C and BCMaMu-C), when evaluated in Caco-2 and HT29-MTX-E12 cells in concentrations ranging from 0.1 to 50 mg/mL. Furthermore, at 0.5 and 1.0 mg/mL, BEMaMu, BCMaMu, and BCMaMu-C prevented the expression of NFKβ induced by TNF-α in Caco-2 cells, while BEMaMu-C and BCMaMu-C activated Nrf2. Therefore, only BCMaMu coated with chitosan (BCMaMU-C) prevented the expression of NFKβ in the presence of TNF-α and activated Nrf2 at 0.5 and 1.0 mg/mL in Caco-2 cells.

## Figures and Tables

**Table 1:** Descriptive analysis of ORAC. Results for different mixtures of CMa and CMu obtained via pre-HPLC.

Samples	N	Average ORAC, µmol ET/100 g	SD	*p*
Exp1. CMa 100% + CMu 0%	3	55,971 ^a^	4940	0.000
Exp2: CMa 50% + CMu 50%	3	53,927	7951	0.999
Exp3: CMa 0% + CMu 100%	3	9918 ^b^	2622	0.000
Exp4: CMa 75% + CMu 25%	3	71,328 ^c^	6221	0.036
Exp5: CMa 50% + CMu 50%	3	36,488 ^d^	6441	0.006
Exp6: CMa 25% + CMu 75%	3	30,438 ^e^	4020	0.001
Optimal mix: CMa 94.5% + CMu 5.5%	3	82,500 ^f^	578	0.000
Total	21	48,653	24,027	

Note: Superscript letters indicate significant differences between experiments. Levene’s test for homogeneity of variances (*p* = 0.172), ANOVA test (*p* < 0.0001), and Tukey’s HSD test were applied at a 5% significance level.

## Data Availability

The data presented in this study are available on request from the corresponding author.

## Abbreviations

Ma	freeze-dried maqui from Isla Natura de Chiloé, Chile.
Mu	freeze-dried murta from Isla Natura de Chiloé, Chile.
C	low-molecular-weight chitosan (Sigma-Aldrich, USA).
EMa	maqui extract obtained with EtOH/H2O (0.1% HCl, *v*/*v*) in an 80/20 *v*/*v* ratio.
EMu	murta extract obtained with EtOH/H2O (0.1% HCl, *v*/*v*) in an 80/20 *v*/*v* ratio.
CMa	maqui concentrate obtained by pre-HPLC.
CMu	murta concentrate obtained by pre-HPLC.
BMaMu	blend of maqui and murta freeze-dried powder in an 80/20 *w*/*w* ratio.
BEMaMu	blend of maqui and murta extract in an 80/20 *w*/*w* ratio.
BCMaMu	blend of maqui and murta concentrate obtained by pre-HPLC in a 94.5/5.5 *w*/*w* ratio.
BEMaMu-C	blend of maqui and murta extract in an 80/20 *w*/*w* ratio coated with a 1% *w*/*v* chitosan solution of acetic acid at a 1.25:1 ratio.
BCMaMu-C	blend of maqui and murta concentrate obtained by pre-HPLC in a 94.5/5.5 *w*/*w* ratio. coated with a 1% *w*/*v* chitosan solution of acetic acid at a 1.25:1 ratio.
